# Chitin Assessment in Insect-Based Products from Reference Methods to Near-Infrared Models

**DOI:** 10.3390/insects16090924

**Published:** 2025-09-02

**Authors:** Audrey Pissard, Sébastien Gofflot, Vincent Baeten, Bernard Lecler, Bénédicte Lorrette, Jean-François Morin, Frederic Debode

**Affiliations:** 1 Knowledge and Valorization of Agricultural Products Department, Walloon Agricultural Research Centre (CRA-W), 5030 Gembloux, Belgiumlecler.craw@gmail.com (B.L.); 2 Ynsect, 91000 Evry, France; benedicte.lorrette@ynsect.com; 3 Eurofins NDSC Alimentaire France, 44323 Nantes, France; jeanfrancois.morin@ftfr.eurofins.com; 4 Life Sciences Department, Walloon Agricultural Research Centre (CRA-W), 5030 Gembloux, Belgium; f.debode@cra.wallonie.be

**Keywords:** insect meal, protein source, NIR spectroscopy, chitin content, chemical composition

## Abstract

The global insect farming sector is rapidly expanding, driven by rising demand for sustainable protein sources and its potential to contribute to food security solutions. This study focuses on the quantification of chitin by comparing two gravimetric methods (ADF-ADL and crude fiber estimation) with a purification method considered as a reference method. It also aims to use the near-infrared spectroscopy (NIRS) to rapidly assess the quality of insect meals, in particular the macronutrients (moisture, protein, fat) and chitin content in a large data set of insect samples. Both alternative methods overestimated chitin content compared to the enzymatic purification method, which is the most reliable but more complex and expensive. Given their advantages (fairly simple, no significant investment, higher sample throughput, relatively short time execution), they can serve for rapid screening when precise chitin determination is not required. Calibration models showed good performance, particularly for protein and fat determination, and satisfactory results for chitin prediction. The NIRS models show promises for rapid and reliable prediction of insect products, although the chitin assessment remains to be further validated. Its implementation could streamline chemical quality control in insect-based food and feed production, offering speed and flexibility for industrial applications.

## Simple Summary

The insect farming sector is growing rapidly due to increasing demand for sustainable protein and its role in improving food security. This study compares two simple gravimetric methods (ADF-ADL and crude fiber estimation) to a purification method, which is considered the reference for measuring chitin. It also investigates the use of near-infrared spectroscopy (NIRS) to quickly evaluate the quality of insect meals, including moisture, protein, fat, and chitin levels, across a large number of samples. The alternative methods tended to overestimate chitin compared to the reference method, which is more accurate but also more complex and costly. However, because they are quick, low-cost, and easy to use, these simpler methods can be useful for rapid screening when high precision is not needed. NIRS calibration models performed well, especially for protein and fat, and gave acceptable results for chitin prediction. NIRS appears to be a promising tool for fast and reliable quality control of insect-based products, although chitin prediction still requires further validation.

## 1. Introduction

An increased interest in insects as an alternative source of protein has been observed during this last decade. Although entomophagy, or eating insects in raw or processed forms (such as protein bars, chips, etc.), has not yet become a habit in European countries [1], the use of insects as animal feed faces less opposition. Due to their composition, insect meals constitute interesting substitutes for ingredients such as soybeans, corn, and fishmeal. Moreover, their production presents many advantages, such as the potential for mass production, low requirements in terms of resources (space, water consumption), and low greenhouse gas emissions, making them more sustainable and environmentally friendly [2,3]. Rearing edible insects has a lower environmental impact compared to producing other animal proteins, specifically poultry, pig, and cattle production [4]. Currently, they are being developed particularly for feeding pigs, poultry, and fish [5], and their possible applications in the field of pet nutrition, especially dogs, have been discussed recently [6]. As mentioned by [7], the European legislation allows eight species to be used for the production of insect meal, i.e., *Acheta domesticus* (Orthoptera, Gryllidae), *Alphitobius diaperinus* (Coleoptera, Tenebrionidae), *Bombyx mori* (Lepidoptera, Bombycidae), *Gryllodes sigillatus* (Orthoptera, Gryllidae), *Gryllus assimilis* (Orthoptera, Gryllidae), *Hermetia illucens* (Diptera, Stratiomyidae), *Musca domestica* (Diptera, Muscidae), and *Tenebrio molitor* (Coleoptera, Tenebrionidae). However, in practice, insect meals intended for animal feed are primarily composed of black soldier fly larvae (*H. illucens*) and yellow mealworm (*T. molitor*).

Insect meals have high nutritional value, as a source of proteins containing essential amino acids, as well as fatty acids, vitamins, and minerals [8,9]. The composition and growth of mealworm larvae, for instance, depend on rearing conditions such as temperature and relative humidity; however, their diet [10,11] also showed a large variability in the nutrient composition of the different larval instars of *Tenebrio molitor*. According to this publication, an earlier larval instar would be a good choice for harvest considering yield, growth rate, and nutritional value.

The primary benefit of insect meals is attributable to their high protein content. However, it is necessary to be cautious when estimating this parameter, as the exoskeleton of insects is composed of chitin, a substance that contains nitrogen and which may be included in the calculation of protein content. Indeed, chitin is composed of β-1,4-linked subunits of N-acetyl-D-glucosamine. It is the second-most-abundant natural polymer after cellulose and a major constituent of insect cuticles [12]. As referenced by [13], chitin is known to have dual effects when included in animal feed: either increasing pathogen resistance by enhancing the innate immune response, or acting as an antinutrient when consumed in high quantities. Therefore, accurate chitin quantification in insect meals would be pertinent. The feed industry is increasingly demanding that chitin content be determined because it contributes to the overestimation of protein content and has the potential to act as an antinutritional factor. The chitin content of insects can be determined using either direct or indirect quantification methods which have been investigated in the past [12,14,15]. Direct methods include the determination of ADF (acid detergent fiber) with or without amino acid correction, crude fiber, and NDF (neutral detergent fiber). Indirect methods include spectrophotometry and ultra-performance liquid chromatography or electrophoretic method [16]. Recently, [13] showed that both the method and the insect life stage have a large impact on chitin determination. According to [12], it has been suggested that fiber in insects could represent chitin because of the similarity of its chemical structure with that of cellulose and because nitrogen was found in the ADF fraction of insects (chitin contains nitrogen atoms). Therefore, ADF content was used for the determination of chitin content in insects. However, chitin rarely exists in a pure form and is usually found in a complex matrix with other compounds, making the extraction of ADF complex. Significant amounts of amino acids were found in the ADF fraction of several insects by [12], showing an overestimation of chitin content when considering ADF only. In 2018, [15] compared three different methods of determining chitin content in insects. The ADF method was used, but ADL (Acid Detergent Lignin) determination was also performed to estimate the ADF-ADL difference. In this way, catechol or quinone-like compounds are removed. In parallel, determination of acetyl groups after total hydrolysis was performed with an enzymatic kit, assuming a degree of acetylation of 90% for chitin in insects. This method assumes that all acetyl groups come from chitin and seems to be the most accurate from an analytical perspective. Results show that ADF content overestimates chitin content, as already mentioned in the literature, whereas ADF-ADL and acetyl group analyses give similar results. Measuring both ADF content and ADL content is therefore a viable alternative to acetyl group analysis for the determination of chitin content.

The use of insects for animal feed is likely to develop more and more in the next decades. According to the latest insights, the market for edible insects for animal feed could extend to USD 2386 million by the end of 2029 [4]. Some companies have already developed and started processing insects in compound feed in Europe, such as PROTIX (Hertogenbosch, The Netherlands), AgriProtein (London, UK), Ynsect (Evry-Courcouronnes, France), and INNOVAFEED (Paris, France). As already mentioned by [7], the use of insect meals as ingredients in animal production require the development, validation, and implementation of analytical tools to ensure nutritional quality. Therefore, it is essential to have rapid and reliable methods to determine their chemical composition. Near-infrared spectroscopy (NIRS), combined with multivariate analysis, presents many advantages. It is a fast and easy technique (minimal sample preparation) for the quantitative determination of several nutritional parameters simultaneously (multi-parameter). It is also considered a green method (it uses little or no chemical reagents), which can be implemented in industry (online analysis) for quality and process control. It has proved to be an effective and reliable method in determining the chemical composition of many food and feed products [17]. Previous studies dealing with NIRS demonstrated the possibility of determining the moisture and protein content [9], as well as the amino and fatty acid composition [11,18], of mealworm larvae, but the chitin content was never investigated. Applying NIRS specifically to chitin determination of insect powder would be a novel contribution to the field, offering a rapid, non-destructive, and cost-effective method for quantifying this important insect component. 

This study aims to evaluate relatively simple gravimetric methods that are suitable for use in resource-limited laboratories, including quality control and process control settings. Ultimately, the goal is to use the gravimetric method as a reference for developing calibration models based on the near-infrared spectroscopy (NIRS), with the aim of predicting the quality of insect meals in a rapid and non-destructive manner.

## 2. Materials and Methods

### 2.1. Chitin Estimation

#### 2.1.1. Insect Samples

For chitin determination, two gravimetric methods were used and compared between two labs (CRA-W and EUROFINS labs) and with a reference method. A set of 20 samples of *Tenebrio molitor* larvae meal was used for this purpose.

#### 2.1.2. Reference Analysis

The reference values (wet chemistry analyses) were realized to determine the chemical composition of the samples. In particular, dry matter, protein, fat, and two methods for chitin estimation based on measuring fibers in the samples were determined. All samples were milled (IKA blender, IKA, Staufen, Germany) prior to analysis to achieve a granulometry below 1 mm. Dry matter content was determined gravimetrically by drying the sample in an oven at 103 °C for four hours. Nitrogen was determined using the Kjeldahl method, with a conversion factor of 6.25 to express nitrogen as protein content [19]. Some discussion remains on the exact value of this conversion factor. Some proposed values for this factor can be found in several works, and it seems to be species dependent [20,21,22]. For fat content, gravimetric extraction was performed with a Soxtec^TM^ 2055 (FOSS, Hilleroed, Denmark) with petroleum ether (40/60).

Several methods have been developed for chitin determination. The advantages and disadvantages have been discussed elsewhere [13,15]. In this work, chitin was estimated using two methods; the first was the Weende method, commonly used for crude fiber (CF) determination [19], and the second involved ADF and ADL determinations as part of the Van Soest method [23]. These two methods are commonly used for proximal composition of animal feed. Since proximate composition demonstrated that fat content was almost always greater than 10%, all samples were first defatted before applying chitin estimation methods (CF or ADF-ADL). The defatting process, which consists of soaking the sample twice for 30 min in petroleum ether (40/60), was performed directly in the crucible used for fiber determination. For CF determination, a homogenized test portion of 1 g was weighed in a filter crucible (porosity P2 40–100 µm). The crucible was then placed in a Fibertec^®^ (Foss, Hilleroed, Denmark). Subsequently, 150 mL of sulphuric acid (0.13 mol·L^−1^) was boiled vigorously for 30 min exactly. Next, the sulfuric solution was removed by filtration and the remaining residue on the crucible was washed three times with 30 mL of boiling water. Then, 150 mL of boiling NaOH solution (0.23 mol·L^−1^) was added and the ebullition was maintained for 30 min. The sodium hydroxide solution was removed by filtration and the residue washed three times with boiling water. Next, the residue was washed with three portions of 25 mL of acetone. The crucible was then dried at 130 °C overnight, cooled, and weighed. It was then transferred into a furnace at room temperature. The furnace was heated to 500 °C and this temperature was maintained for three hours. After cooling, the crucible was placed in a crystallizing dish and weighed again once it had completely cooled. For ADF determination, a homogenized test portion of 1 g was weighed in a filter crucible (porosity P2 40–100 µm). The crucible was then placed in a Fibertech^®^ (Foss, Hilleroed, Denmark) and 100 mL of 20 g L^−1^ of cetyltrimethylammonium bromide (CTAB) in 0.5 Mol L^−1^ H_2_SO_4_ was added to the sample. Then, the obtained suspension was boiled for one hour. The acid reagent was removed by filtration and the remaining sample in the crucible was rinsed with boiling water until neutrality was reached. The crucible was then washed with 2 × 20 mL of acetone and placed in an oven overnight at 103 °C. After a cooling phase in a crystallizing dish, the crucible was weighed. For ADL measurement, the crucibles used to obtain the ADF were placed in a crystallizing dish containing 72% (*w*/*w*) sulfuric acid with the remaining sample. The suspension was stirred with glass rods, and any lumps were broken up with the same rod. After three hours, sulfuric acid was removed under vacuum filtration and rinsed with boiling water until neutrality was reached. The crucible was then washed with 2 × 20 mL of acetone and placed in an oven overnight at 103 °C. After a cooling phase in a crystallizing dish, the crucible was weighed. Finally, the crucible was placed in a muffle furnace and the ambient temperature was set to 525 °C, which was maintained for one hour for mineralization of the remaining sample. After a cooling step, the crucibles were placed in a crystallizing dish and weighed once they had completely cooled.

These two methods were compared with a laboratory scale method for chitin purification. This purification method was considered as the reference for chitin measurement. It is based on three specific extractions, adapted from the patent (US-11958918-B2). This procedure, which was specially adapted for *Tenebrio molitor* cuticles, makes it possible to achieve a chitin purification higher than 98%. It was shown that the purified chitin contains a small fraction of residual amino acids, but no more lipids. The operating procedure consists of three strong steps to purify chitin via enzymatic and chemical methods. Unlike fiber methods, the test sample size is deliberately large to ensure greater homogeneity and reproducibility; indeed, 50 g of insect meal is extracted via these three steps. One enzymatic extraction is first performed with a protease (alcalase) at 60 °C for 24 h. After this extraction step, the reactive medium is heated to 95 °C to deactivate enzymes. The residue obtained after filtration is purified a second time using sodium hydroxide (1 M) for five hours at 95 °C. The residue is then filtered and extracted a third time with sodium hydroxide (1 M) for 16 h at 95 °C to ensure a full purification of the chitin. The final residue is rinsed with distilled water until a neutral pH is achieved and dried in an oven for 24 h. The residue weight divided by the initial amount of the insect meal (50 g) is considered as the percentage of chitin in the sample. All results are expressed on dry matter basis (DM).

### 2.2. Near-Infrared Spectroscopy

#### 2.2.1. Spectra Collection

Different types of insect-based samples (in particular dried and ground insects) and insect-based products (diverse processed products from both the food and feed industries) were used to build a database. The ground insects included adults or larvae of different species. The database included the set of 20 samples of *Tenebrio molitor* larvae meal used for the chitin estimation. This latter species was highly represented in different forms in the database, along with other kinds of species such as *Hermetia illucens*, *Blabtica dubia*, *Locusta migratoria*, *Acheta domesticus*, *Zophobas morio*, *Gryllodes sigillatus*, *Gryllus assimilis*, and *Alphitobius diaperinus*.

A total of 154 samples were studied. They were scanned using an XDS spectrometer (FOSS NIRSystems, Inc., Silver Spring, MD, USA) covering the visible and NIRS range from 408 nm to 2498 nm. The samples were scanned twice, and the spectra were averaged for statistical analyses.

#### 2.2.2. Preprocessing and Multivariate Analysis

Spectra and reference values (moisture, protein, fat, CF, and ADF-ADL content) were used to build spectroscopic models using WinISI software Version 4.10.0.15326 (Infrasoft International LLC, State College, PA, USA), using the spectral range ranging from 1100 to 2498 nm. Different pretreatments, such as first derivative, SNV Detrend and first derivative, none, were applied to the raw spectra, and calibration models for the different chemical parameters were developed using the Partial Least Squares (PLS) regression technique. The first derivative is used to enhance subtle differences in spectra, highlight peak positions, and help resolve overlapping spectral features while SNV Detrend (Standard Normal Variate with Detrending) corrects for spectral variations caused by factors like light scattering and baseline shifts. It helps to remove multiplicative and additive effects, making the spectra more comparable and easier to interpret. To evaluate the predictive potential of NIRS models, the database was split into two sets: a calibration set to develop the calibration models, and a validation set to check the performance of the models. The split was realized randomly based on the composition content of the samples; in particular, according to the protein, fat, and fiber contents, such that there were two sets with the same composition and variability. The accuracy of the calibration models was evaluated based on the following statistics: coefficient of determination of calibration (R^2^c), root mean square error of calibration (RMSEC), the coefficient of determination of cross-validation (R^2^cv), and the root mean square error of cross-validation (RMSECV).The accuracy of the validation step was evaluated based on the coefficient of determination of prediction (R^2^p) and the root mean square error of prediction (RMSEP). The samples included in the validation set were not totally independent from the ones included in the calibration set. They differ from the samples included in the calibration set, but some samples may come from the same batches or the same providers. Ratios of performance to deviation (RPD), which is the ratio between the standard deviation and the root mean square error of the calibration/validation, were also calculated to evaluate the predictive ability of the models. According to [24], an RPD value between 1.5 and 2.0 indicates the ability to distinguish between high and low values, 2.0 to 2.5 allows for approximate quantitative predictions, 2.5 to 3.0 suggests a good prediction, and above 3.0 indicates excellent prediction.

## 3. Results

### 3.1. Chitin Estimation

For chitin determination, two alternative gravimetric methods were used and compared between two labs (Lab_01 and Lab_02). For this purpose, a set of 20 samples was analyzed by applying the CF method and the ADF-ADL method. All results were compared with the chitin purification method which was used as the reference one (Table 1). Normality of data sets was assessed by using the Shapiro–Wilk test. The *p*-values obtained for the difference in each paired data set are presented in Table 2 and suggest that data are likely normally distributed.

**Figure 1:**
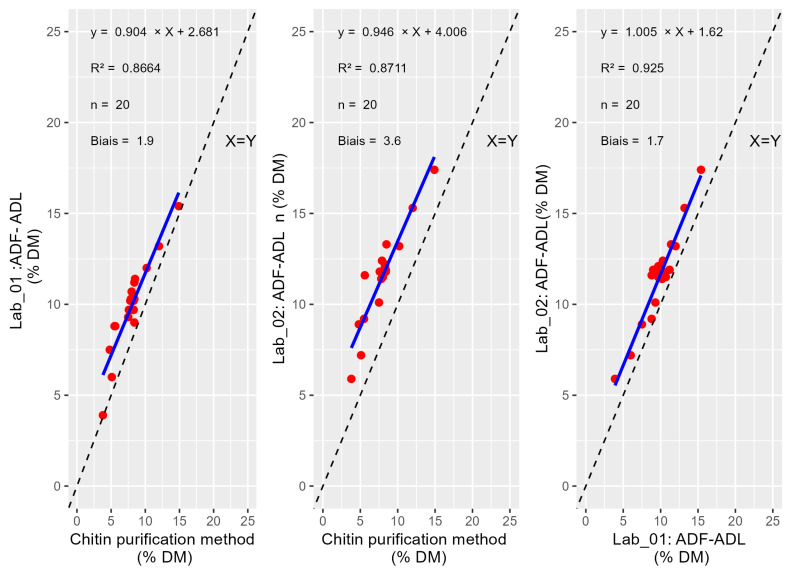
Comparison of methods and labs based on ADF-ADL values.

**Figure 2:**
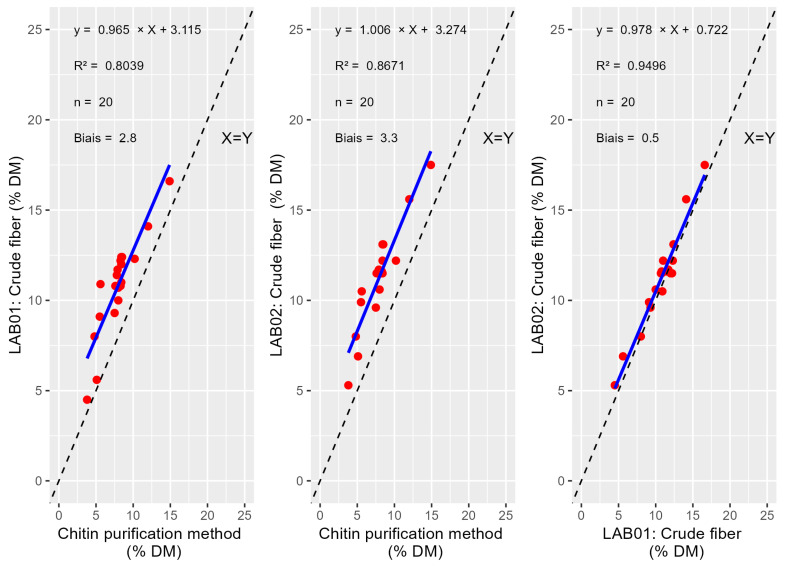
Comparison of methods and labs based on CF values.

The obtained correlation for ADF-ADL between purification methods and gravimetric methods are presented below (Figure 1). The same data treatment was realized for CF (Figure 2). The T-tests for paired values show significant bias between reference method and Lab_01 and Lab_02 for the ADF-ADL content (*p*-values = 1.56 × 10^−8^ and 3.73 × 10^−13^) respectively and between the labs (*p*-value = 1.61 × 10^−9^) (Table 3). The T-tests for paired values show significant bias between reference method and Lab_01 and Lab_02 for CF (*p*-value = 1.87 × 10^−9^ and 4.76 × 10^−12^) respectively and between the labs (*p*-value = 0.002).

The comparison of the three methods revealed statistically significant biases among the different testing conditions. Paired T-tests demonstrated significant differences in the results for ADF-ADL measurements between the purification method and the two laboratory methods (Lab_01 and Lab_02), with *p*-values of 1.56 × 10^−8^ and 3.73 × 10^−13^ respectively. These *p*-values indicate a strong likelihood that the observed differences are not due to random variation, suggesting a consistent systematic positive bias between the reference method and both laboratory protocols. This can be attributed to the purification process employed in the reference method, which enables exhaustive purification of the chitin. In contrast, the two gravimetric methods—which were originally developed for plant-based matrices—are less specific, as they used only chemical treatments rather than specific enzymatic degradation. Moreover, a significant bias was also observed between the two laboratory methods themselves (*p*-value = 1.61 × 10^−9^), highlighting potential methodological discrepancies even within controlled lab environments. These findings suggest that the choice of analytical method can substantially affect the results and underscore the need for method harmonization or calibration when comparing data across platforms.

The paired T-test analysis revealed statistically significant biases in CF measurements among the different methods assessed. In particular, significant differences were observed between the purification method and both laboratory methods: Lab_01 (*p*-value = 1.87 × 10^−9^) and Lab_02 (*p*-value = 4.76 × 10^−12^). These very low *p*-values indicate systematic differences rather than random variation, suggesting that laboratory protocols yield results that consistently deviate from the purification method. Additionally, a significant bias was found between Lab_01 and Lab_02 (*p*-value = 0.002), pointing to inter-laboratory variability. While this difference is less than the one involving the reference method, it nonetheless underscores the need for careful calibration and method standardization across different analytical settings. These results highlight the importance of methodological harmonization, particularly when comparing or integrating data from different sources. Two additional species, *Hermetia illucens* (black soldier larvae) and *Gryllus assimilis* (adult), were evaluated using the three methods. As shown in Table 4, the results were comparable to those obtained with *Tenebrio molitor* larvae. To be more reliable for other species, a larger number of samples must be taken into account and confirm the fact that gravimetric method overestimates chitin compared to the purification method.

Tested methods based on fibers analysis (CF and ADF-ADL) are both biased compared to the purification method. These biases are known and constant between methods and labs. This allows for value correction for external or internal values in the case of quality control. They are also able to provide valuable data for the development of NIRS methods. From an implementation standpoint, these gravimetric methods are fairly simple, do not require significant investment, and offer higher sample throughput. Their execution time is also relatively short; 2 to 3 days for ADF-ADL and about 1.5 days for CF, compared to the chitin purification method, which takes around 4 to 5 days to complete. Finally, only a small amount of material is required for the implementation of the two gravimetric methods. Indeed, only 1 g is needed per test, compared to 50 g in the case of the purification method. This represents a significant advantage, particularly when experimental material is available in small quantities, such as in the early stages of process development.

### 3.2. Near-Infrared Spectroscopy Analyses

#### 3.2.1. NIRS Spectra

**Figure 3:**
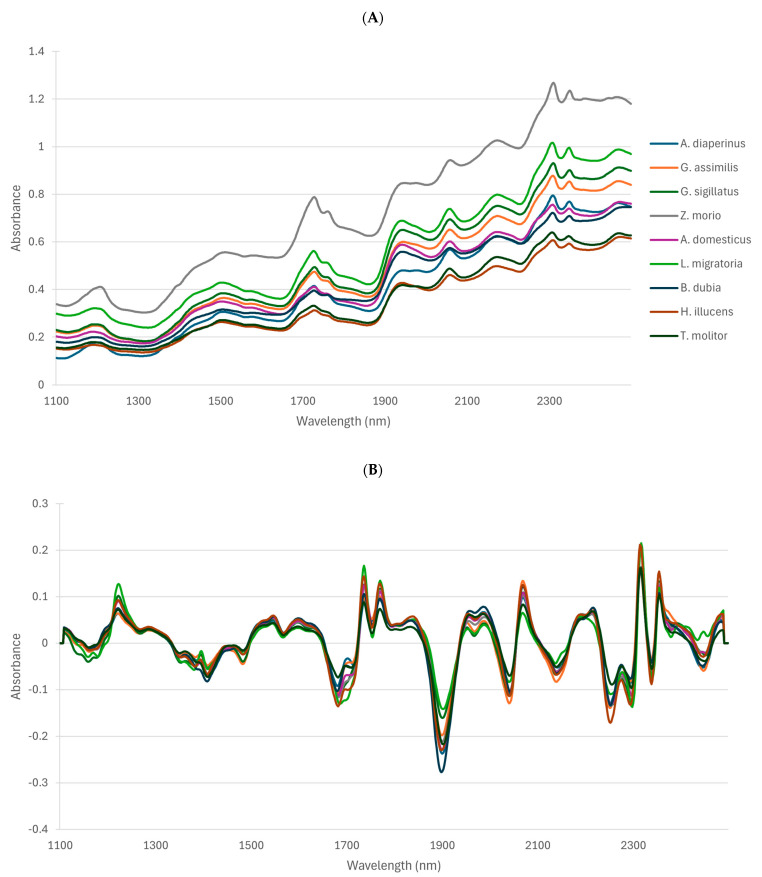
Raw NIRS spectra (**A**) and SNVD1 preprocessed spectra (**B**) of the different species analyzed in this study.

Figure 3 represents the raw absorbance spectra (A) and the preprocessed spectra (B) of different insect species (*Tenebrio molitor*, *Hermetia illucens*, *Blabtica dubia*, *Locusta migratoria*, *Acheta domesticus*, *Zophobas morio*, *Gryllodes sigillatus*, *Gryllus assimilis*, and *Alphitobius diaperinus*) analyzed in this study in the 1100–2500 nm range. Major absorbance bands may be observed in the different NIRS regions. The same main bands that were identified previously in [25] may be observed at about 1200, 1728, 1760, 1940, 2056, 2172, 2306, and 2348 nm. These have been related to the major organic compounds, i.e., water, protein, and fat content. However, the peaks at around 1200, 1510, 2050, and 2180 nm could also be related to the chitin constituent. Indeed, [26] measured squid pen and red snow crab shell, which are raw materials of chitin and chitosan, and showed these four typical peaks in the NIRS spectra. According to [27,28], spectral bands at 1200, 1510, and 2050 nm may be associated with second overtone C-H stretching, first overtone N-H stretching, and N-H stretching + amide II, respectively.

Differences in absorption between species may be observed in intensity (absorbance values), but some differences appear also in the wavelength domain, which may be related to differences in chemical composition. In actuality, the content of protein, fat, and fibers (chitin), but also the different kinds of these molecules, i.e., different types of proteins and lipids, may vary among species.

#### 3.2.2. Calibration Results

The data were split into a calibration set (in accordance with the parameter, the set includes between 88 and 128 samples) and a validation set (in accordance with the parameter, the set includes between 20 and 26 samples). The number of samples, the mean, and the standard deviation of both sets are shown in Table 5.

As shown, not all samples were characterized for fat content; only 88 and 20 samples from the calibration and validation sets, respectively, were analyzed for this parameter. Likewise, the moisture and fiber content were known for only 100 samples from the calibration set.

The division of the samples into the calibration and validation sets was realized to maintain the same variability in both sets as far as possible. The extreme reference values were included in the calibration set to contain the maximum variability. The calibration set was used to develop models with PLS regression. The performance of the calibration models obtained with the different pretreatments of the spectra is presented in Table 6.

For all parameters, some outliers (“H” outliers based on Mahalanobis distance measurements and “T” outliers based on *t* criteria) were removed during the development of the calibrations, so that the number of samples in the models differed from that of the calibration set. With respect to the SECV, R^2^cv, and RPD values, models showed variable calibration accuracy among the different spectral preprocessing. Calibration models without any pretreatment yielded the lowest accuracy. Mathematical preprocessing of the spectra made it possible to increase the accuracy of the calibration models. As revealed by the highest RPD and R^2^cv, and the lowest SECV values, the most effective models were obtained using the SNV Detrend and first derivative for all parameters. It is likely that this pre-treatment enhances the identification of the spectral features truly related to the chemical composition of interest and the signal-to-noise ratio facilitating analysis and modeling. Therefore, these models will be used for the validation step.

High coefficients of determination and low errors of cross-validation (SECV) were observed for moisture, protein, and fat content, giving rise to high values of RPDcv (higher than three) and indicating good performances of the models. Considering the best spectral pretreatment (SNV Detrend and first derivative), a RPDcv value of 6.9 was observed for protein content, which reached 8.7 for fat content. These high values of RPDcv suggest a high degree of accuracy of the calibration models. For the models related to CF and ADF-ADL content, the RPDcv values were slightly lower, with a value of around three.

The samples from the validation set were predicted using the calibration models. The performance of the validation set is shown in Table 7. The moisture content showed particularly high accuracy, with a very low SEP giving rise to a very high RPDp value. For the other parameters, the same trends may be observed compared to the cross-validation results. For protein and fat content, validation results showed similar values compared to the calibration step. Standard errors of prediction (SEP) were similar, or slightly higher, to the errors of cross-validation (SECV). High values of RPDp were obtained, as observed in cross-validation (RPDcv). For CF and ADF-ADL content, accuracy was slightly lower, with lower coefficients of determination and lower RPDp values (between 2 and 2.5).

**Figure 4:**
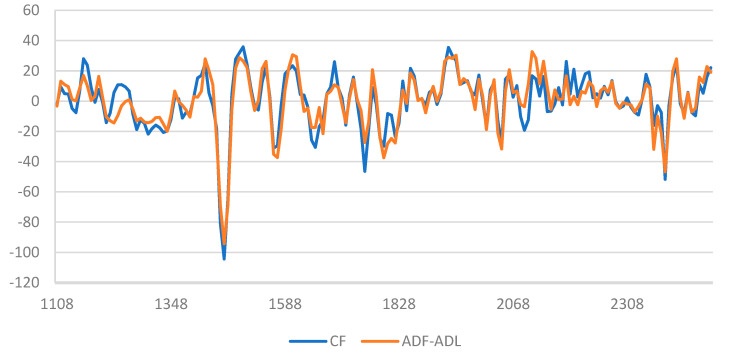
Beta coefficients of calibration models for CF and ADF-ADL content obtained with the best mathematical pretreatment of the spectra (SVND-D1).

The beta coefficients of the best preprocessed models for CF and ADF-ADL regression models are presented in Figure 4. It should be noted that, except for some shifts, the coefficients are similar for both constituents. This suggests that the chemical information related to the prediction of CF and ADF-ADL content is similar. Numerous peaks may be observed, and the most important wavelength may be highlighted at 1164, 1244, 1460, 1500, 1756, 1932, 2388, and 2412 nm. Ref. [29] reported 16 important wavelengths to predict CF in his study. As he mentioned, CF does not completely represent chitin, which is mixed with other molecules to constitute the total crude fiber. Ref. [30] reported that the region between 2000 and 2500 nm is associated with chitin. Here, in our study, we observed two peaks in this region which are involved in the prediction of CF and ADF-ADL content and are thus potentially related to chitin calibration. Ref. [31] showed the possibility of analyzing the degree of deacetylation (DD) of chitinous materials using NIRS and achieved good performance. They indicated that characteristic wavelengths for DD evaluations appeared between 1200 and 1300 nm, where the second overtone of C-H bond occurs, and they obtained the best MLR models with four wavelengths, with a specific band around 1246 nm.

## 4. Discussion

### 4.1. Chitin Estimation

In this work, two alternative methods to the traditional chitin extraction/purification process (which takes four to five days) were tested, i.e., the ADF-ADL method and the CF method. The ADF-ADL method applied by the two labs overestimates chitin content in comparison to the results obtained with the extraction/purification method. Based on the CF determination method, as for ADF-ADL, both labs overestimated the chitin content in comparison to the chitin purification/extraction approach. It was concluded that the extraction–purification method is probably the most accurate method for chitin determination. However, it is time-consuming and must be used for precise chitin determination or as a pilot process to obtain pure chitin. Alternative methods, such as ADF-ADL or the CF method could be used for a rapid estimation of chitin content, even if they overestimate chitin values. It means that these methods can be used for process assessment (ranking between batches for example), but not for an accurate chitin determination. The CF method is the fastest, with an analysis time of 1.5 days, followed by ADF-ADL (2 to 3 days), and finally the purification–extraction method, which requires at least 4 days. The analysis time could still be significantly reduced. This could be achieved thanks to the development of NIRS approaches and adequate models.

### 4.2. Use of NIRS for Macronutrient Predictions

The chemical composition of insect meals varies according to the species, but also according to the substrate used for rearing them [32]. It has also been demonstrated that the chemical composition varies according to the developmental stages (egg, larva, pupa, adult) [8,13]. In this study, we collected a large number of insect-based samples, including samples belonging to different species, different development stages (larva or adult), and, undoubtedly, samples coming from different rearing conditions. This explains the wide variability observed in terms of chemical composition. Indeed, samples showed a very large range for protein content (17–82%), fat content (1.5–41%), and chitin estimated by CF content (0.1–17%).

The performance of the calibration models obtained in the present paper suggested that NIR spectroscopy can be used to predict the macronutrient content in insect-based products. They may be compared to previous similar studies dealing with edible insects. Ref. [9] analyzed mealworm larvae with NIRS. As observed here, they obtained calibration models with better predictive accuracy using preprocessed spectra (compared to raw spectra) and showed that the best calibration models for moisture and protein content were obtained using the first derivative spectra. High-performing calibrations were obtained for both parameters. Compared to our results, the error of prediction for protein content was lower (RMSEP = 0.508); however, the RPD value was similar (RPD = 4.13). The study of [11] focused on the prediction of fat and fatty acid contents in living *Tenebrio molitor* larvae. Regarding the fat content prediction, they attained a very low error of prediction (RMSEP = 0.28) and a high RPD value (RPD = 8.33). As discussed in [25], the performance of calibrations may vary according to the set-up and the material investigated. The particularity of our study was to build a very large database of diversified insect-based products and not to focus on one, or a reduced number of, species. As the validation was realized on relatively small numbers of samples (especially for fat content due to availability of the data), further analyses could be carried out to validate the performance of the models.

Previous research using handheld NIRS devices has also been conducted to predict the macronutrient content of insect powders [33,34]. Ref. [33] tested two handheld FT-NIR devices (NeoSpectra MDK and NeoSpectra Scanner, Si-Ware Systems, Inc., Cairo, Egypt) on samples of *Tenebrio molitor* (mealworm, *T. molitor*) and *Alphitobius diaperinus* (lesser mealworm, *A. diaperinus*) for fat content determination. They developed PLSR models with Neospectra Scanner spectra, reaching RPDcv values between 7.42 and 10.09, and showed the potential of using handheld FT-NIR devices in conjunction with multivariate analysis to rapidly quantify the fat content in insect powders. Ref. [34] used two different portable NIR spectrometers (SCiO, Consumer Physics, and NeoSpectra Micro Development Kit, Si-Ware) to predict the macronutrient content (proteins, fatty acids, carbohydrates, fibers) in five commercially available insect powder samples. The PLS calibrations showed RPD values ranging from 1.8 to 3.8 for the Neospectra, and from 3.65 to 11.01 for the SCiO. They concluded that both instruments were useful for classifying different insect powders, regardless of their provenance or grinding degree, and had the potential to be used in the quality control phases in the insect food industry.

In this study, in addition to the common macronutrients (moisture, protein, and fat contents), the development of calibration models for determining chitin content was attempted. Two methodologies were followed to chemically estimate the chitin content, and calibration models were developed with values coming from both methods. Prediction results showed satisfactory results with RPDp values of 2.4 and 2.1 for CF and ADF-ADL content, respectively. In terms of RPD, models with values between 2 and 2.5 are generally considered as moderate quality models. It means that, in industrial applications, the models will not provide accurate quantitative results but only rough estimates. Therefore, at the present time, the models for CF and ADF-ADL may be used for sorting samples into categories (e.g., high vs. low content) and provide a rough screening. Where precise quantification is required (e.g., for quality control), chemical methods should be used. Ref. [35] assessed the ability of NIRS to evaluate the ADF content of BSFL instars (fifth and sixth) and obtained good cross-validation results (R^2^cv = 0.90; RPDcv = 3.6). To our knowledge, this is the first study where chitin content is indirectly predicted by NIRS in insect powder samples. Application of near-infrared spectroscopy for chitin determination is thus innovative and may contribute to new industrial applications offering a rapid, non-destructive, and cost-effective method for predicting this important insect component. Additional investigation could be carried out to improve the prediction performances and to obtain quantitative models. Indeed, the development of an accurate and rapid method to quantify chitin is relevant for many industrial uses. Its presence in food and feed can have important impacts, including negative impacts. Chitin is known as an antinutritional factor, since it can have negative effects on digestion in certain contexts by interfering with the absorption of other nutrients. An excess concentration of chitin in feed can be counterproductive [15]. On the other hand, recently, a few authors [36,37,38] have examined the characteristics and properties of chitin and chitosan from insects (*T. molitor, H. Illucens*) and demonstrated their similarity with the commercial products derived from crustaceans, suggesting that insects could represent novel and interesting sources of these biopolymers. Ref. [39] published a review focusing on their sources, production methods, characterization, physical and chemical properties, and the potential applications in diverse domains, including emerging biomedical applications.

### 4.3. Implementation of NIRS in Industry

NIRS has been applied in the food industry for process and quality control for a long time. It could also be integrated into the rearing process to ensure the chemical composition of insect-based products and insect powders in a rapid and easy way, accelerating quality control in the emerging insect food and feed industries. Implementation of calibration models such as those developed here in industry or laboratory workflow could be achieved by calibration transfer, which involves techniques to apply a calibration model developed on one instrument (the “primary” or “master” instrument) to another instrument (“secondary”) or under different conditions. It may require some effort to enable a calibration model to be effectively transferred between two instruments. However, the calibration transfer could save time and resources, as the development of robust calibrations is very costly and time-consuming (mainly due to the reference method analyses). Finally, once models have been transferred, they can be used directly by any kind of user. Various standardization and preprocessing methods have been developed for calibration transfer of NIRS spectra, which has been largely documented [10,40,41,42].

Furthermore, technological advances in recent decades have led to the development of small and handheld NIRS devices. They provide a very useful solution for on-site applications, especially in situations where measurements are needed without the need for extensive sample preparation or traditional lab equipment. Recently, their suitability in the context of insect protein production has been investigated. Ref. [34] demonstrated the usefulness of two handheld devices for classification and showed good performances of calibration (prediction of macronutrient content). Recently, [43] showed the potential of one device to assess multi-species insect protein adulteration. In this study, the NIRS approach was investigated using a benchtop instrument only. As previously realized with another agricultural product [44], we could attempt calibration transfer to handheld instruments in order to propose a compact and ready-to-use device for chemical quality and process control in insect-farming industries.

## Figures and Tables

**Table 1:** Comparison between the reference method (chitin purification method) and the two gravimetric methods (ADF-ADL and CF) used in two labs (Lab_01 and Lab_02) for a set of 20 samples. All results are expressed on a dry matter basis (DM).

	Reference Method	Lab_01		Lab_02	
*Tenebrio* *molitor* Sample	Chitin (% DM)	ADF-ADL (% DM)	CF (% DM)	ADF-ADL (% DM)	CF (% DM)
Sample_01	5.6	8.8	10.9	11.6	10.5
Sample_02	8.5	11.4	12.4	13.3	13.1
Sample_03	8.3	9.7	12.2	12.1	11.5
Sample_04	8.4	10.3	12.0	11.8	11.5
Sample_05	8.3	10.2	10.8	12.2	11.6
Sample_06	7.9	10.3	11.7	12.4	11.7
Sample_07	7.6	9.7	10.8	11.8	11.5
Sample_08	7.8	10.2	11.4	11.4	11.6
Sample_09	8.0	9.7	10.7	11.5	11.5
Sample_10	8.4	9.0	11.0	11.9	12.2
Sample_11	3.8	3.9	4.5	5.9	5.3
Sample_12	5.1	6.0	5.6	7.2	6.9
Sample_13	7.5	9.3	9.3	10.1	9.6
Sample_14	10.2	12.0	12.3	13.2	12.2
Sample_15	12.0	13.2	14.1	15.3	15.6
Sample_16	14.9	15.4	16.6	17.4	17.5
Sample_17	8.4	11.2	12.4	11.9	13.1
Sample_18	4.8	7.5	8.0	8.9	8.0
Sample_19	8.0	10.7	10.0	11.5	10.6
Sample_20	5.5	8.8	9.1	9.2	9.9
n	20	20	20	20	20
Mean	8.0	9.9	10.8	11.5	11.3
Min	3.8	3.9	4.5	5.9	5.3
Max	14.9	15.4	16.6	17.4	17.5
Range	11.1	11.5	12.1	11.5	12.2

**Table 2:** *p*-values of Shapiro–Wilk tests between the methods in two labs (Lab_01 and Lab_02).

	Reference Method	ADF-ADL_Lab_01	CF_Lab_01	ADF-ADL_Lab_02	CF-Lab_02
Reference method	x	0.5985	0.6276	0.2778	0.6059
ADF-ADL_lab_01		x	0.9781	0.5554	0.6191
CF_Lab_01			x	0.0927	0.3613
ADF-ADL_Lab_02				x	0.3665
CF-Lab_02					x

**Table 3:** *p*-values of paired T-tests between reference and gravimetric methods.

	Reference Method	ADF-ADL_Lab_01	CF_Lab_01	ADF-ADL_Lab_02	CF-Lab_02
Reference method	x	1.556 × 10^−8^	1.871 × 10^−9^	3.737 × 10^−13^	4.762 × 10^−12^
ADF-ADL_lab_01		x	6.469 × 10^−5^	1.607 × 10^−9^	1.685 × 10^−7^
CF_Lab_01			x	2.183 × 10^−5^	2.220 × 10^−3^
ADF-ADL_Lab_02				x	6.632 × 10^−2^
CF-Lab_02					x

**Table 4:** Results obtained on two different insect species.

	Reference Method	Lab_01		Lab_02	
*Species*	Chitin (% DM)	ADF-ADL (%DM)	CF (%DM)	ADF-ADL (%DM)	CF (%DM)
*Hermetia illucens*	5.4	8.6	7.4	8.3	6.9
*Gryllus assimilis*	5.8	7.9	10.1	10.8	10.1

**Table 5:** Moisture (moist), protein (prot), fat (fat), CF (cell), and ADF-ADL content (expressed in % as_is) for calibration and validation sets.

	Calibration Set					Validation Set				
	Moist (%)	Prot (%)	Fat (%)	Cell (%)	ADF-ADL (%)	Moist (%)	Prot (%)	Fat (%)	Cell (%)	ADF-ADL (%)
N	100	128	88	100	100	26	26	20	26	26
Mean	4.75	59.90	16.05	8.25	8.20	4.70	59.21	18.22	9.39	9.24
Minimum	1.50	16.59	1.56	0.1	0.09	1.70	47.38	4.56	5.98	5.2
Maximum	10.31	81.90	40.96	17.05	16.96	8.28	74.07	33.99	14.06	14.97
SD	1.93	12.10	9.77	3.72	3.82	1.74	8.63	8.08	2.52	2.79

N = number of samples; SD = standard deviation.

**Table 6:** Calibration models for moisture (moist), protein (prot), fat (fat), CF (cell), and ADF-ADL content (expressed in % as_is) obtained using PLS regression with different mathematical pretreatment of the spectra.

Constituent	Pretreatment	N	SD	LVs	SEC	R^2^c	SECV	R^2^cv	RPDcv
Moist (%)	None	89	1.81	7	0.41	0.95	0.48	0.93	3.8
	D1	87	1.85	8	0.34	0.96	0.48	0.93	3.9
	SNVD-D1	87	1.86	8	0.18	0.99	0.39	0.95	4.8
Prot (%)	None	115	8.98	6	2.17	0.94	2.23	0.94	4.0
	D1	116	9.65	6	1.78	0.96	2.39	0.94	4.0
	SNVD-D1	120	10.96	8	1.20	0.99	1.59	0.98	6.9
Fat (%)	None	83	8.86	8	1.07	0.98	1.26	0.98	7.0
	D1	81	8.68	5	0.82	0.99	1.00	0.99	8.7
	SNVD-D1	80	8.68	6	0.78	0.99	1.00	0.99	8.7
Cell (%)	None	95	3.46	8	1.67	0.77	1.89	0.70	1.8
	D1	97	3.57	8	0.92	0.93	1.23	0.88	2.9
	SNVD-D1	97	3.63	8	0.82	0.95	1.22	0.88	3.0
ADF-ADL (%)	None	92	3.50	8	1.55	0.80	2.10	0.66	1.7
	D1	92	3.55	8	0.91	0.93	1.38	0.85	2.6
	SNVD-D1	93	3.64	8	0.80	0.95	1.13	0.90	3.2

N = number of samples in the calibration; SD = standard deviation; LV = latent variables; SEC = standard error of calibration; R^2^c = coefficient of determination of the calibration; SECV = standard error of cross-validation; R^2^cv = coefficient of determination of cross-validation; RPDcv = ratio of performance deviation = SD/SECV. D1 = 1st derivative; SNVD-D1 = standard normal variate with detrending and 1st derivative.

**Table 7:** Validation results for moisture (moist), protein (prot), fat (fat), CF (cell), and ADF-ADL content (expressed in % as_is).

	N Val	SD	SEP	R^2^p	RPDp
Moist (%)	26	1.74	0.19	0.99	9.2
Prot (%)	26	8.63	1.53	0.97	5.6
Fat (%)	20	8.08	1.44	0.97	5.6
Cell (%)	26	2.52	1.03	0.83	2.4
ADF-ADL (%)	26	2.79	1.32	0.77	2.1

N Val = number of samples in the validation; SEP = standard error of prediction; R^2^p = coefficient of determination of prediction; RPDp = ratio of performance deviation = SD/SEP.

## Data Availability

The original contributions presented in this study are included in the article. Further inquiries can be directed to the corresponding author.
